# Analysis of Influencing Factors of Urban Community Function Loss in China under Flood Disaster Based on Social Network Analysis Model

**DOI:** 10.3390/ijerph191711094

**Published:** 2022-09-05

**Authors:** Lianlong Ma, Dong Huang, Xinyu Jiang, Xiaozhou Huang

**Affiliations:** 1 College of Public Administration, Huazhong University of Science and Technology, Wuhan 430074, China; 2 School of Management, Wuhan University of Technology, Wuhan 430070, China; 3 School of Statistics and Mathematics, Hubei University of Economics, Wuhan 430205, China

**Keywords:** flood disaster, social network analysis, urban community, functional loss, influencing factors

## Abstract

The increasing frequency of floods is causing an increasing impact on urban communities. To identify the key influencing factors of functional loss in Chinese urban communities under floods, this paper explored the influencing factors and factor combinations through a social network analysis approach using the 265 cases of urban communities in China affected by floods collected from 2017–2021 as research data. The key influencing factors and factor combinations were identified comprehensively using multiple indicator analyses such as core-periphery structure, node centrality, and factor pairing. The analysis results showed that “road disruption”, “housing inundation”, and “power interruption” are the three most critical factors affecting the functional loss of urban communities in China under floods, followed by “residents trapped”, “enterprises flooded”, and “silt accumulation”. In addition, “road disruption–housing inundation”, “housing inundation–residents trapped”, and “road disruption–residents trapped” are the most common combinations of influencing factors.

## 1. Introduction

Floods are considered to be the most devastating common meteorological hazard in the world [1,2]. In the past 20 years (2000–2019), global flood losses have reached approximately USD 651 billion [3]. Moreover, the frequency, impact, and risk of floods are still increasing worldwide [4,5,6,7]. China is one of the countries most severely affected by floods [1,8]. In the past three decades (1984–2018), the annual average flood loss in China reached USD 19.2 billion, accounting for 54% of the total direct economic loss due to climate disasters in China [9]. More than half of the population and two-thirds of the land area of China are affected by floods every year [10,11]. Meanwhile, rapid urbanization increases the risk of urban flooding [12,13,14]. More than half of the global population lived in cities in 2019, and China’s urbanization rate is expected to exceed 70% in 2050 [15]. It is an urgent and realistic issue to study the impact of flood disasters on urban communities in order to reduce disaster losses and enhance people’s well-being.

The concept of resilience and its relevance to disaster risk management has received increasing attention in recent years [16]. Resilience has been used in a variety of disciplines including ecology, physics, engineering, and social sciences to explain the responses of individuals, organizations, societies, ecology, physical systems, and communities to certain disturbances [17]. It is now seen as a desirable characteristic of structures, infrastructure, and communities [18,19,20]. Reducing the vulnerability of urban systems and reducing the risk of cascading failures in the face of climate change and related disasters is one of the key elements in building urban resilience [21,22]. Infrastructure, in turn, is the interface between a city’s “material social well-being” and socio-economic activities and the built environment, playing a key role in ensuring the resilience and sustainability of urban communities [23,24]. Therefore, the scope and definition of infrastructure now gradually takes on broad characteristics and can be defined as any asset or system that provides services or delivers goods to social citizens [25]. It includes both traditional public infrastructure (such as electricity, gas, water, communication and transportation systems) and social infrastructure (such as emergency medical services or food supply chains) [25].

Floods can cause not only damage to physical infrastructure, but also more severe losses and ripple effects or even long-term impacts on society due to the disruption of critical infrastructure services and their interconnectedness [26,27]. In addition, the social consequences of infrastructure failures often greatly outweigh their physical damage [23]. Some recent studies have noted linking infrastructure disruptions to social impacts [28,29]. However, these studies mainly use infrastructure damage as the initial context for cascading failures, without considering the initial context in which multiple community functions may be affected simultaneously in a nonsequential manner in the disaster context. It has not been possible to answer the question “What are the most important resources for a community in the event of a flood?”. As such, this is the central concern of this paper.

The ability to provide critical resources or services to a community is directly related to its survival during a disaster and recovery afterwards [16]. Community function is a manifestation of community resilience [30]. Based on the understanding of the concepts of resilience and generalized infrastructure, this paper defines community functions in the context of disasters as the services provided by communities to maintain the normal operation of community residents, environment, infrastructure, and other systems under disaster disturbances. The interdependence of failing infrastructure systems and limited resources make it impossible for all community functions or components to be protected at all times [28,31]. It is important to identify the critical services that need to be prioritized during a disaster and the linkages between them to ensure that community functions are able to maintain at least minimum operations without complete disruption. Therefore, the focus of this paper is on how to use the degree of correlation of systems to identify the key influencing factors of community function loss under flooding, so as to minimize community vulnerability and cascade effects, and thus improve urban community resilience.

## 2. Materials and Methods

### 2.1. Social Network Analysis Model

Social network analysis (SNA) is a method to study a group of participants and their relationships, which has been widely used in political science, management, psychology, and sociology studies [32,33,34,35,36]. In recent years, it has gradually emerged in the field of disaster and environment. As the SNA model can summarize all factors and their relationships into a network, and analyze both nodes in the network and their relationships [37], it is very suitable for analyzing the realistic situation in which influencing factors of community function loss often occur together in this study. In addition, the importance of influencing factors under the SNA model comes from the association with other factors, which provides a new perspective for factor importance identification studies [37]. Therefore, in this study, the SNA model was chosen to comprehensively analyze the factors influencing the loss of community functions under floods and the relationships between them.

The SNA model consists of two parts: the nodes and the lines connecting them [38]. The nodes are the variables selected by the researcher and the influencing factors in this paper. The line connecting the nodes is the relationship between the nodes. There are two types of lines: undirected lines that do not take into account the sequential relationship of nodes, and directed lines that take into account the sequential relationship of nodes. The focus of this paper is on the co-occurrence of factors, not on the sequence of factors, so we choose the undirected line. The types of social network models are divided into 1-mode and 2-mode, with 1-mode social networks focusing on the internal relationships of nodes and 2-mode social networks focusing on the relationships between two different types of nodes [38]. In this paper, the influencing factors of interest are at the same level, so the 1-model social network is chosen for analysis. As the analysis focus of this paper is on the influencing factors rather than the density comparison or network evolution trend of different networks, the analysis of nodes in this paper considered core/peripheral structure analysis and centrality analysis. Meanwhile, the co-occurrence matrix was used to identify the combination of influential factors with high co-occurrence frequency.

**Figure 1:**
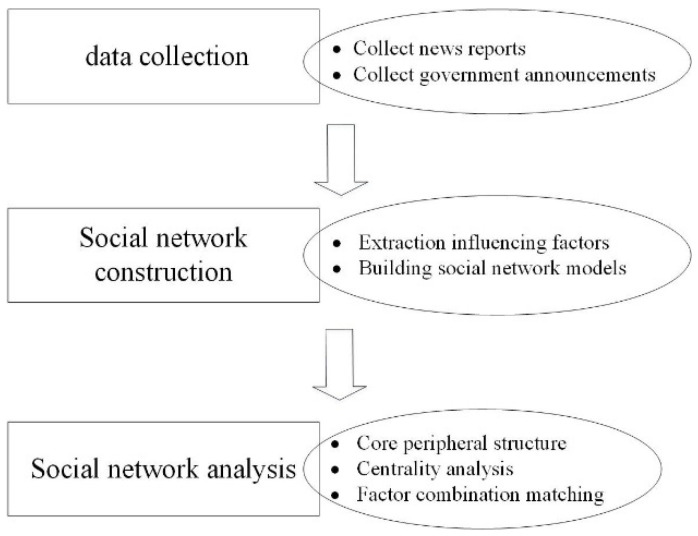
The research flow chart.

Therefore, the research process of this paper had three stages: data collection, model construction, and indicator analysis. In the first stage, we mainly used news media reports to collect raw data. In the stage of model construction, text analysis was carried out on the original data to extract all the influencing factors that can be identified from the text. In addition, the social network model was constructed by establishing an adjacency matrix. In the stage of the index analysis, core/peripheral structure analysis, centrality analysis, and factor pairing analysis were used to identify the core influencing factors and their combination. The research flow chart is shown in Figure 1.

### 2.2. Data Collection

Community-level disaster loss data are too microscopic to be directly available in national statistics. Therefore, the data in this paper were extracted from Internet resources such as authoritative news media reports, official policy statements (e.g., post-disaster recovery policies, recognition summaries, etc.). Nowadays, more scholars have adopted this way of collecting initial data from the Internet to conduct their research [25,26,39].

The specific collection process is as follows: Firstly, we used the advanced search function of the Baidu search engine to search. The search terms “community + flood” were selected to record and filter all media reports on floods that occurred in the five years from 2017 to 2021 at the community level. Secondly, we eliminated media reports that did not give key information. The selection was based on the ability to provide clear community names, geographic locations, time of occurrence, and effective coverage that included disaster factors. The process was completed by manual identification, and the identification standards referred to the US Department of Homeland Security (DHS) designation for critical infrastructure industries [28]. Factors present in key infrastructure implementation industries were identified. Finally, 265 cases of Chinese urban communities affected by floods were recorded. Some of the recorded information is shown in Table 1.

### 2.3. Model Construction

#### 2.3.1. Initial Extraction of Influencing Factors

The initial text data needs to be further mined to extract the influencing factors before it can be used for social network analysis. In this paper, we used case study and text recognition methods to encode and analyze the text data to extract the influencing factors. Firstly, key statements related to the influencing factors of community function loss were coded into representative tags. For example, as mentioned in the report of “Chengnan Road Community”, “Chengnan Road community is close to The Xiong’er River and was seriously affected by the rainstorm and waterlogging disaster, with river collapse, road subsidence and mudstone blocking everywhere in the area”. In this original sentence, three influencing factors can be extracted, which are “river landslide”, “road subsidence” and “mudstone blocking”. Then, the extracted labels were summarized and sorted. For example, labels such as “water in power pipes”, “damage to power facilities”, “power system failure”, “cut off power to the house” and “power interruption to the power department” were summarized as “power interruption”. Finally, 28 influencing factors of community function loss under flood disaster were summarized, as shown in Table 2.

#### 2.3.2. Construction Process

In this paper, we first constructed a 2-mode social network of “affected community-influencing factors”. That is, a 265 × 28 matrix A*_xy_* was constructed, with columns *x* (*x* = 1, 2, …, 265) representing affected communities and rows *y* (*y* = 1, 2, …, 28) representing influencing factors. They corresponded to the 265 community cases collected above and the 28 influencing factors compiled from them, respectively. In the matrix, binary values were taken. If factor *y* appears among the factors influencing the loss of function of a community *x*, then the matrix cell a(*x*,*y*) will take the value 1, otherwise it will take the value 0.

Second, we converted the 2-mode social network into a 1-mode social network. The principle of this step is to construct a 28 × 28 matrix B*_yy_*, where B = A^T^A (A^T^ is the transpose of matrix A above). In other words, matrix B can be obtained by multiplying the “transpose of matrix A” with “matrix A”. Matrix A is the matrix that forms the 2-mode network in the above paragraph. This conversion step was mainly done by the software UCINET 6.689 (Analytic Technologies, Lexington, KY, USA). The full name of UCINET is “University of California at Irvine Network”, which is the most popular social network analysis software at present. It is developed by Steve Borgatti, Martin Everett and Lin Freeman and the program is distributed by Analytic Tech-nologies which located in PO Box 910359, Lexington, KY 40513 USA. By clicking on Affiliation (2-mode to 1-mode) in the Data option, the transformed 1-mode network of “influencing factors-influencing factors” was obtained, which can be used for the comprehensive analysis of influencing factors. The rows and columns of matrix B are the influencing factors, and the numbers of the matrix cells represent the frequency of the influencing factors together.

### 2.4. Indicator Analysis

Core-periphery structure analysis can distinguish core nodes from edge nodes as a whole. Core nodes are often closely related to other nodes in the network and occupy an important position [40]. As such, the core community function loss influencing factors tend to be more closely linked to each other. Node pairing analysis can identify the combination of influencing factors that often occur together [37]. That is, the co-occurrence frequency of the influencing factors of community function loss can be analyzed. Centrality is an indicator to evaluate the importance of a node in the overall network structure, which is also the most commonly used analysis method [41]. There are a number of centrality indicators with different emphasis on importance. Among them, degree centrality, closeness centrality, and betweenness centrality are the three most common ones [41].

Degree centrality is defined as the number of connections of a node (the number of lines connected to that node in the network) [42]. It focuses on measuring the frequency of connections between nodes, and its value is proportional to the importance of the nodes. The influencing factor with a higher degree centrality is the most frequently associated with other influencing factors among all the factors that affect community function.

The mathematical expression for the degree centrality of a node *i* is:

(1) $$D\left(i\right)=\underset{j}{\sum }d_{ij}$$

(2) $$D_{s}\left(i\right)=D\left(i\right)/\left(n-1\right)$$

where Equation (1) is the absolute degree centrality, *j* represents the nodes other than *i*. If there is a connection between node *i* and node *j*, then *d_ij_* = 1, otherwise it is 0. Equation (2) is the relative degree centrality, which is the degree centrality after normalization and is more reasonable and accurate, where *n* is the number of nodes in the social network, and the same below.

Betweenness centrality can be defined as the number of node pairs connected by a given node [42]. It is an indicator that defines importance in terms of the number of times a node needs a given node to reach another node [43]. It focuses on the degree of control of communication between nodes, and its value is also proportional to the importance of the node. The greater the betweenness centrality of the influencing factor, the more it controls for co-occurrence relationships among other influencing factors of community function loss.

The mathematical expression for the betweenness centrality of a given node *i* is as follows:

(3) $$B\left(i\right)=\underset{p,q}{\sum }B_{piq}/B_{pq}$$

(4) $$B_{s}\left(i\right)=2B\left(i\right)/\left(n^{2}-3n+2\right)$$

In Formula (3), *B*(*i*) is the absolute betweenness centrality. Among them, *B_pq_* refers to the number of shortest paths from node *p* to node *q*, *p* and *q* are two different nodes other than node *i*, and *B_piq_* refers to the number of shortest paths that need to pass through node *i* from node *p* to node *q*. *B*_s_(*i*) in Equation (4) is the relative betweenness centrality after normalization.

Closeness centrality refers to the total distance between a node and all other nodes in the network [43]. It focuses on measuring the connection distance between nodes [42], so its value is inversely proportional to the importance of the nodes. Therefore, we often normalize it to measure it positively. After standardization, the influence factors with greater closeness centrality have a faster co-occurrence relationship with other influence factors of community function loss.

(5) $$C\left(i\right)=\underset{i}{\sum }c_{ij}$$

(6) $$C_{s}\left(i\right)=\left(n-1\right)/C\left(i\right)$$

The mathematical expression for the closeness centrality of a given node *i* is Equation (5). Its normalized mathematical expression is shown in Equation (6).

## 3. Results and Discussion

### 3.1. Core-Periphery Analysis

**Figure 2:**
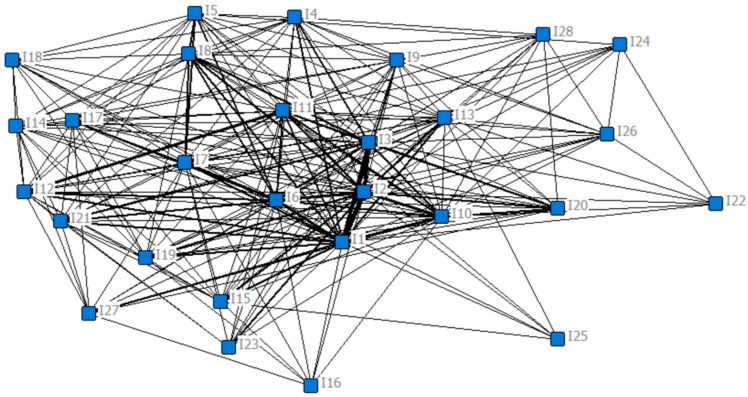
The social network of influencing factors of Chinese urban community function loss under flood disaster.

Two results were obtained from the core/periphery structure analysis with the software UCINET 6.689. First, the network concentration index of the “influencing factor-influencing factor “1 model social network constructed above is 84.07%, which is a relatively high value. This means that the network as a whole may have only one core region. Further, the 28 nodes were divided into two categories: core nodes and periphery nodes. The 28 nodes correspond to the 28 influencing factors in Table 2 above by serial number. There are five core nodes, namely “road disruption” (I_1_), “housing inundation” (I_2_), “residents trapped” (I_3_), “power disruption” (I_6_), and “enterprises flooded” (I_10_). The rest are periphery nodes. Meanwhile, in order to visualize the analysis results more intuitively from the whole, the 1 model social network was visualized using NetDraw (a component of UCINET 6.689). As shown in Figure 2, the social network consists of 28 nodes and 470 lines. The 470 lines represent the 470 co-occurrence relationships between the nodes. It is clear from Figure 2 that the social network has very dense connections at the center and relatively sparse connections at the edges. Moreover, I_1_, I_2_, I_3_, I_6_ and I_10_ are in the center of the dense connection lines.

### 3.2. Centrality Analysis

**Figure 3:**
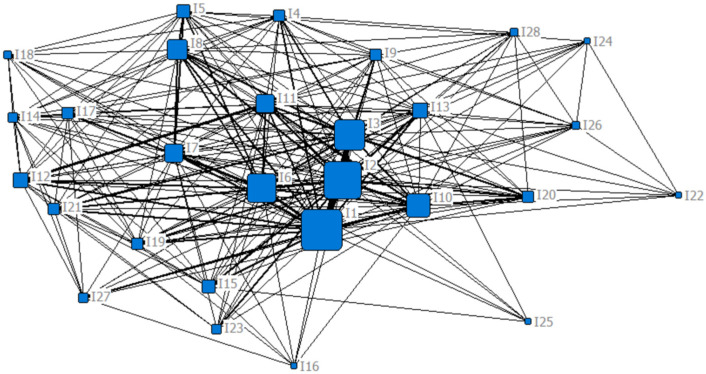
The social network of the influencing factors of Chinese urban community function loss under the flood disaster with the added degree centrality attribute.

**Figure 4:**
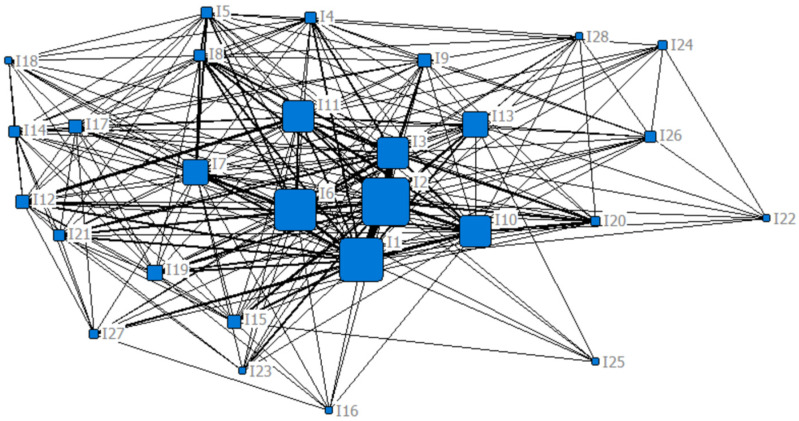
The social network of the influencing factors of Chinese urban community function loss under the flood disaster with the added betweenness centrality attribute.

**Figure 5:**
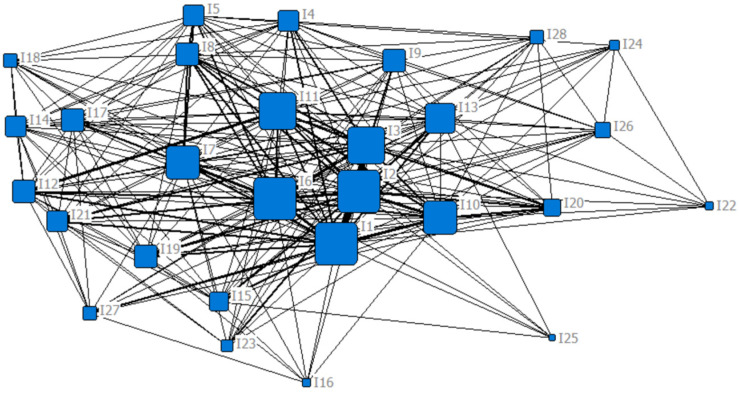
The social network of the influencing factors of Chinese urban community function loss under the flood disaster with the added closeness centrality attribute.

Figure 3, Figure 4 and Figure 5 shows the social network of influencing factors of Chinese urban community function loss under flood disaster after adding degree centrality, betweenness centrality, and closeness centrality, respectively. The larger node shape means the greater centrality of the node.

Meanwhile, based on the number of core nodes, this paper focused on the top five influencing factors, which were displayed in Table 3. The degree centrality, betweenness centrality, and closeness centrality were calculated for each factor using Equations (2), (4) and (6), respectively. It can be observed that the ranking of the top five influencing factors is different, but the differences are small.

#### 3.2.1. Degree Centrality

The top five influencing factors of degree centrality were “road disruption” (I_1_), “housing inundation” (I_2_), “residents trapped” (I_3_), “power interruption” (I_6_), and “enterprise flooded” (I_10_). “Road disruption” was the factor with the highest degree centrality, understandably, as too much water on the road can slow down all rescue operations, which can trigger other influencing factors. “Housing inundation” and “residents trapped” were ranked second and third, respectively. Floods not only cause flooding of roads, but also easily enter low-rise households or buildings in low-lying areas. If residents do not evacuate in advance, they may not be able to escape independently if their homes are extensively flooded, and may even be at risk to their lives in the event of water fall. “Power interruption” was ranked fourth. It can be seen that, also belonging to the energy infrastructure, the interruption of power supply is more troublesome to the normal life of residents than the interruption of water supply and gas supply. This is probably due to the fact that electricity is the source of power for household appliances and public utilities in modern life. When there is an interruption in power supply, everything from public services in the external environment (e.g., lights, medical services, etc.) to electrical facilities in the internal environment (e.g., elevators, household appliances) are affected. The last influencing factor in the top five of degree centrality is “enterprises flooded”. On the one hand, this may be because community-based enterprises such as small supermarkets and vegetable shops are the providers of daily meals for many families. The flooding of these service-oriented businesses will cause a lot of disruption to the normal life of the community. On the other hand, the flooding of larger manufacturing enterprises may be more problematic. If the equipment or raw materials of these enterprises are flooded, the disaster will have a ripple effect due to the industrial connection, which will lead to greater economic losses.

#### 3.2.2. Betweenness Centrality

The top five influencing factors of betweenness centrality were “housing inundation” (I_2_), “road disruption” (I_1_), “power interruption” (I_6_), “enterprise flooded” (I_10_), and “silt accumulation” (I_11_). “Housing inundation” (I_2_) was in first place. Housing is the last barrier for residents against flooding. If the flood is blocked outside the house, the loss caused by other factors will not cause serious injuries or damage for the individual residents in a short time. Otherwise, it can quickly lead to bad consequences, such as residents being trapped or emergency power outages to prevent electrocution safety. Therefore, “housing inundation” has the strongest control over the other factors. “Road disruption” came next. Roads are the first infrastructure to begin to bear the brunt of flooding. In the same way, if the road can drain the flood water as soon as possible, it will not lead to the occurrence of other impacts. Otherwise, the water from the road will penetrate into other buildings or damage other facilities, triggering other impacts to occur together. As the rain increases the conductivity of electricity, electrical systems are often actively or passively disrupted during flooding for the safety of residents. This makes the betweenness centrality of the “power interruption” also relatively high. The subsequent “enterprise flooded” may be due to the continuous direct and indirect influence of businesses on the maintenance and recovery of residents’ lives. It is also an influencing factor that is in a strong control. At last, the reason for the high betweenness centrality of the “silt accumulation” may be that it is a barrier that must be overcome to restore normal community function.

#### 3.2.3. Closeness Centrality

In the closeness centrality ranking, “road disruption” (I_1_), “housing inundation” (I_2_), and “power interruption” (I_6_) were tied for first. It can be seen that housing, roads, and electricity are all infrastructure that are closely related to the lives of residents. Moreover, these infrastructures are so interconnected that the destruction or failure of one of them will have an immediate cascading effect. The disruption of the road may lead to water entering a house or electrical equipment failing due to water overflow in a short time. The transmission between them is direct and rapid. As such, these three influencing factors are relatively close to each other. This is the reason for the high closeness centrality of these three factors. “Residents trapped” and “silt accumulation” were tied for second place. The high frequency of road disruptions due to flooding makes it easy to understand that residents are often trapped. In addition, silt almost always appears in different degrees with floods, and it is not difficult to occur together with other influencing factors.

In summary, “road disruption”, “housing inundation”, and “power interruption” were in the top four of the three centrality categories. “Residents trapped” appeared in the third place of degree centrality and tied for the second place of closeness centrality. “Enterprises flooded” appeared in fifth place of degree centrality and fourth place for betweenness centrality. “Silt accumulation” appeared in fifth place for betweenness centrality and tied for second place for closeness centrality.

The results of the core-periphery analysis and the above three centrality analyses were combined. From the three aspects of association frequency, control, and proximity, we can conclude that “road disruption”, “housing inundation”, and “power interruption” are the three most critical influencing factors of Chinese urban community function loss under flooding. If the second most critical influencing factors also need to be noted, they are “residents trapped”, “enterprises flooded”, and “silt accumulation”.

### 3.3. Factor Pairing Analysis

The following analysis focused on the top three “two-factor combinations” in terms of co-occurrence frequency. They were identified from the adjacency matrix of the “influencing factor–influencing factor” 1-model social network constructed in the previous section.

“Road disruption–housing inundation” was the most likely “two-factor combination” to occur simultaneously. They appeared together 43 times, accounting for 16.23% of the total number of events. This indicates that in urban flooding, road disruption often occurs together with housing flooding. Urbanization has been proven to increase the frequency of flooding [44]. Overused concrete and asphalt have created impermeable surfaces that cannot absorb water, which is one of the reasons for frequent urban flooding [45]. In order to have enough housing environment to accommodate the rapidly growing population migrating from rural to urban areas, cities tend to remove natural rainwater-retaining infrastructure such as green spaces, woodlands, wetlands, and natural lakes [45]. This means that areas that were used to store natural rainwater may be used for residential or living purposes. Therefore, “road disruption” and “housing inundation” often occur simultaneously.

The combination of “housing inundation–residents trapped” ranked the second most frequent co-occurrence. Together, they appeared 33 times, accounting for 12.45% of the total number of events. According to the collected textual data, not all residents are willing to accept the warning and leave their houses at the beginning of the flooding. The adoption of protective responses by individual residents varies from person to person depending on individual flood experience, flood knowledge, vulnerability, risk perception, and risk communication [46,47,48]. Some theories suggest that individual’s high risk perception needs to be accompanied by effective response assessment to generate protective responses [46]. Therefore, it is not easy for residents to proactively take protective responses, which often leads to residents who do not take protective responses in advance to be trapped in the event of a flood because they are too late to evacuate. Another reason may be that the evacuation of a large number of people in a short period of time has high requirements on the capacity of government emergency personnel and the resources of shelters [49,50].

The combination of “road disruption–residents trapped” appeared together 31 times, accounting for 11.7% of the total number of events, and was ranked third. Residents’ subjective willingness to go out is related to whether the roads are smooth. Residents’ success in getting out is related to the severity of the road disruption. When roads are disrupted, residents’ mobility is greatly restricted. As roads are flooded, it becomes difficult and dangerous for residents to go out. For their own safety, residents’ willingness to move is subjectively affected by road disruptions. At the same time, the government and media will inform residents to reduce their travel for safety. Additionally, when roads are significantly flooded, residents may already be surrounded by floodwater and cannot move out freely under objective conditions. Therefore, due to the subjective aspect of residents’ willingness to go out and the objective aspect of the severity of road disruption, “road disruption” and “residents trapped” often occur together.

## 4. Conclusions

This study aimed to identify the key influencing factors of functional loss in Chinese urban communities under flooding. Using the research method of social network analysis, through core-periphery analysis, centrality analysis, and factor matching analysis, the influencing factors were comprehensively analyzed from the perspectives of the whole, individual, and local social network.

In terms of overall factors, the results of the core-periphery analysis showed that “road disruption”, “housing inundation”, “residents trapped”, “power interruption”, and “enterprises flooded” are the core factors among all the factors. In terms of individual factors, “road disruption”, “housing inundation”, and “power interruption” were identified as the three most critical factors through the combination of degree centrality, betweenness centrality, and closeness centrality. The second most critical influencing factors are “residents trapped”, “enterprises flooded”, and “silt accumulation”. The top three “two-factor combinations” with the highest frequency of co-occurrence were identified by factor pairing analysis, namely “road disruption–housing inundation”, “housing inundation–residents trapped”, and “road disruption–residents trapped”. Through these analysis results, the influencing factors and co-occurrence combination priorities of urban community function loss in China under flood disaster can be determined. It is beneficial to reduce community losses and recover quickly, which is very useful in building community resilience.

Based on the above conclusions, we summarized several recommendations on prevention and adaptation measures for urban communities in response to flood disasters. Protecting critical infrastructure, including lifeline systems, remains the primary option for governments to keep communities functioning properly. First and foremost, full cycle protection of the road system is paramount. For example, when flooding is not occurring, road maintenance and upgrades should receive attention to improve permeability. When flooding occurs, the government should quickly organize efforts to dredge the sewers and reduce water on the roads. When the flooding is over, the cleaning of silt and garbage on the roads also needs to get the attention of the government, which is also important for the restoration of community functions. Secondly, in the post-disaster recovery phase, the government can give priority to the restoration of the electricity system. Electricity has the most critical impact on community function in the energy system. In addition, the prevention of “housing inundation” and “residents trapped” requires the joint efforts of the government and residents. Residents are not only victims of flooding, but also an important force in coping with flooding. This requires a greater willingness to prepare for disasters and an increase in preparedness behaviors. For example, evacuating before the disaster or stocking up on household sandbags to avoid “housing inundation” and “residents trapped”.

In addition, since “enterprises flooded” is also a core factor, it will affect the food supply of residents. The government should pay enough attention to the issue of stocking emergency supplies and supplying them quickly. Finally, based on the results of factor pairing analysis, when one of the influencing factors occurs, the other factor should be given maximum protection. In this way, the co-occurrence phenomenon between factors is blocked. For example, when roads are severely flooded, the government’s first priority may be to evacuate residents who are about to be trapped or rescue those who are already trapped to reduce the damage caused by the disaster.

This study also has some limitations. The first is that the types of communities are not classified. Only urban communities are considered, but not rural communities. In China, different types of communities can lead to differences in the resources they receive. For example, rural communities have much poorer access to infrastructure services than urban communities. There will also be differences in emergency response forces and the natural environment. This makes it possible that the factors influencing the loss of community function under flooding may differ depending on the community type. Meanwhile, the influence of geographic and temporal factors of the city where the community is located is not considered in the urban community; for example, the difference between a central city and a remote city, and the difference between a new community and an old community. Secondly, the hazard of the disaster is not analyzed specifically. Different intensity of flooding may cause different depths of urban inundation, and the threat to community functions should be different. These two aspects can be studied in more depth in the future.

## Figures and Tables

**Table 1:** Cases of flood-affected urban communities in China, 2017–2021 (partial).

No.	Name	Address	Time	Source URL
1	Rong Yuan Community	Huizi District, Zhengzhou City, Henan Province	20 July 2021	https://baijiahao.baidu.com/s?id=1706348093553095716andwfr=spiderandfor=pc (accessed on 4 March 2022)
2	Zhenghua Community	Jinshui District, Zhengzhou City, Henan Province	22 July 2021	http://yjj.henan.gov.cn/2021/10-21/2331497.htmlnews/6713.html (accessed on 4 March 2022)
-	-	-	-	-
265	Tao Li Yuan Community	Hongjiang District, Huaihua City, Hunan Province	29 June 2017	https://news.sina.com.cn/o/2017-07-15/doc-ifyiamif3027345.shtml (accessed on 15 May 2022)

**Table 2:** Influencing factors of urban community function loss in China under flood disaster.

No.	Influencing Factors	No.	Influencing Factors
1	Road disruption	15	Crop destruction
2	Housing inundation	16	Landslide
3	Residents trapped	17	Gas supply interruption
4	Residents panic	18	Elevator interruption
5	Underground garage flooded	19	Drainage failure
6	Power interruption	20	Residents refused to evacuate
7	Water supply interruption	21	Vehicles flooded
8	Food and drinking water shortages	22	Greenery destruction
9	Medical services disrupted	23	Home appliances soaked
10	Enterprises flooded	24	Senior services disrupted
11	Silt accumulation	25	Crowd gathered
12	Garbage accumulation	26	Community offices flooded
13	Embankment failure	27	Rescuers injured
14	Communication interruption	28	School closed

**Table 3:** Values and rankings of the top five factors of the three centrality attributes.

Id	*D_s_*(*i*)	Ranking	Id	*B_s_*(*i*)	Ranking	Id	*C_s_*(*i*)	Ranking
I_1_	0.2188	1	I_2_	0.053	1	I_1_	0.9643	1
I_2_	0.199	2	I_1_	0.0489	2	I_2_	0.9643	1
I_3_	0.149	3	I_6_	0.0447	3	I_6_	0.9643	1
I_6_	0.137	4	I_10_	0.0327	4	I_3_	0.9	2
I_10_	0.1085	5	I_11_	0.0311	5	I_11_	0.9	2

## Data Availability

The datasets used and analyzed during the current study are available from the corresponding author on reasonable request.
